# Investigating the landscape of US orphan product approvals

**DOI:** 10.1186/s13023-018-0930-3

**Published:** 2018-10-22

**Authors:** Kathleen L. Miller, Michael Lanthier

**Affiliations:** 1 Office of Orphan Products Development, Office of the Commissioner, US Food and Drug Administration, 10903 New Hampshire Ave, Silver Spring, MD 20903 USA; 2 Office of Planning, Office of the Commissioner, US Food and Drug Administration, Silver Spring, USA

**Keywords:** Orphan drug act, Orphan products, FDA

## Abstract

**Background:**

The Orphan Drug Act was enacted in 1983 to encourage the development of drugs for rare diseases. Previous research has attempted to examine the impact of the Act by assessing either the number of orphan designations that have been granted or the number of new orphan drugs approved for marketing. This study provides a more in-depth understanding of the effect of the Orphan Drug Act by investigating all types of drug approvals with an orphan designation, along with multiple characteristics of the drugs, over the entire 35 years of the Act. These orphan approvals include: new molecular entities (new drugs approved first for a rare disease), secondary indications (an expansion from the first approved indication), and new formulations.

**Results:**

The results show that the number of approvals for orphan indications has been increasing over time, and the upward trend is especially large in the most recent years. Much of this increase has been driven by the increase in secondary indications being approved for previously approved drugs, although there have also been increases in the number of approved new drugs. We also find that while oncology indications have been increasing significantly, there has also been an increase in other therapeutic areas. Additionally, we find that the proportion of biologic drugs being approved has increased over time. Lastly, while other parts of this drug landscape have dramatically altered over time, the proportion of orphan approvals receiving priority review has not changed.

**Conclusions:**

Our data suggest that the Orphan Drug Act appears to have stimulated significant drug development for rare diseases. Additionally, approvals of orphan indications have been increasing over time. This increasing effect has not targeted a single area of the rare disease space, rather, gains in approvals have been seen across: therapeutic areas, approval types (both new drugs and secondary indications), and for both biologics and small molecule drugs.

**Electronic supplementary material:**

The online version of this article (10.1186/s13023-018-0930-3) contains supplementary material, which is available to authorized users.

## Background

The Orphan Drug Act (“ODA” or “the Act”) was enacted in 1983 to encourage the development of drugs for rare diseases. Prior to this legislation, few drugs had been approved for rare conditions, and advocates were concerned that market forces would not be enough to induce further development. The ODA created financial incentives, such as tax credits on clinical trial expenses and a seven-year exclusivity period for orphan approvals, with the goal of having many new treatments developed, approved, and made available for patients [1, 2]. In order to receive these benefits, the drug must be intended to treat a rare disease, defined, in the US, as affecting fewer than 200,000 people [2]. If it is, and all designation criteria are met, the company developing the drug can apply for and receive an orphan designation.

Several studies have analyzed the overall effects of this legislation [3]. Most have focused on the number of orphan drug designations granted over time, and found that designations have been increasing [4–8]. These authors suggest that their results indicate that the ODA has been successful in stimulating new development, and the presence of more designations leads to more therapies being available. Additionally, another study investigated the number of orphan new molecular entities (NMEs) that had been approved since the legislation was enacted [9]. The authors found that the number of approved orphan NMEs has increased over time, and this was especially true in oncology.

While studying the number of orphan designations and orphan NME approvals provides insight into the success of the ODA, it does not examine the full effects of the legislation. Therefore, in this study, we analyze all types of approvals with orphan drug designations (NMEs, secondary indication approvals on approved drugs, and new formulations of approved drugs) from 1983—2017. By investigating the full scope of approvals, we can obtain a more complete picture of the impact of the ODA. Additionally, the approval of new formulations has implications for increased patient adherence and reduced treatment burden, while the number of secondary indication approvals has important implications for patient reimbursement and access, as well as important safety and effectiveness information for patients and providers [10].

We also investigate multiple characteristics of these approvals to more fully characterize the effects of the ODA on the output of new therapies for patients with rare diseases. These include: the therapeutic area the drugs were approved to treat, whether they were biologics or small molecule drugs, and whether the drugs received a priority review from the FDA.

Understanding the therapeutic areas of the approved drugs may help policymakers determine whether the effects of the Act are skewing towards any single group: if most approvals are occurring in a single therapeutic area, it is possible that additional policies could be put into place to target development in other areas.

Understanding the distribution of biologic versus small molecule drugs has significant implications for future generic entry. New biologic drugs receive twelve years of regulatory exclusivity, while new small-molecule drugs receive five years. (These are the respective new compound exclusivities; patent exclusivities and other regulatory exclusives may extend the effective market exclusivity periods of these drugs.) Because these drugs are regulated under different pathways, the timing of entry of generics or biosimilars will be different. This could have future effects on pricing and patient access.

Lastly, understanding the number of drugs that have received a priority review provides an insight into one measure of whether these orphan drugs represent a significant improvement relative to available therapies.

## Methods

The primary dataset for this analysis was extracted from the public “Orphan Drug Product Designations and Approvals” searchable list, which is maintained by the Office of Orphan Products Development at the US Food and Drug Administration (FDA) [11]. This searchable list contains all drugs that have been granted an orphan designation for a specific indication, and further indicates whether the drug has received marketing approval by the FDA for the orphan indication.

For this analysis, we define the unit of observation as the drug-indication pairing. This is because there are multiple types of drug approvals, which will be discussed further in the next paragraph. All drug-indication pairings that received both an orphan designation and an FDA marketing approval were extracted and aggregated into a dataset (The final dataset is provided in the Additional file 1: Appendix). Additional drug-level characteristics were pulled from an internal regulatory database, as well as from the public Drugs@FDA database [12].

The following characteristics were identified for this analysis. First, we identify the type of regulatory approval that the orphan designated molecule-indication received:

1. *Orphan NME approval.* The first time a molecule, “drug”, is approved for use in the US, we call the approval a new molecular entity, or NME. The NME is approved for a specific indication, for which safety and efficacy studies have been completed and reviewed by the FDA

For example, the drug Opdivo (nivolumab) was approved as an NME in 2014, for the specific orphan indication “the treatment of patients with unresectable or metastatic melanoma and disease progression following ipilimumab and, if BRAF V600 mutation positive, a BRAF inhibitor”.

2. *Orphan secondary indication.* Once a drug has been approved, for either a rare or non-rare indication, it is possible that new indications can be also approved for the drug. These subsequent indications can include either of the following:
  1. indications for a different disease than the first approved indication, in other words, a new population/indication, such as Hodgkin lymphoma when the first indication was metastatic melanoma; or,
  2. indications related to the first approved indication, in other words, an extension within a population/indication, such as an extension to a pediatric population within the same disease.
    We call all these subsequent indications “secondary indications”.
    For example, Opdivo (nivolumab) was approved for what we classify as a secondary indication in 2016 for “the treatment classical Hodgkin lymphoma that has relapsed or progressed after autologous hematopoietic stem cell transplantation (HSCT) and post-transplantation brentuximab vedotin”.
3. *Orphan new formulation.* Once a drug has been approved for use, for either a rare or non-rare indication, it is also possible for new formulations of the drug to be approved

This category of approvals may include cases where, for example, a drug that was approved for intravenous administration is reformulated to be used as an oral liquid (this is also known as a change in the route of administration).

Second, for drugs approved between 1993—2017, we identify whether the drug received a priority review or a standard review. The priority review designation program began in 1992, and allows for an expedited FDA review of drugs that are for serious or life-threatening diseases and have the potential to be a significant improvement over existing therapies. Drugs that are designated priority review have an FDA review timeline goal of six months, rather than the ten-month standard review. While there were a few drugs approved under this paradigm in 1992, we begin our analysis in 1993 to start at a complete year of data. We have not been able to identify the priority review classification of five orphan approvals that occurred during this period.

Third, we identify whether a drug was a small molecule or a biologic. We define a biologic as a product that was produced by a biological expression system that had been manipulated using techniques such as genetic engineering, cell fusion, or other technologies. This includes products that are regulated by both the Center for Biologics Evaluation and Research and the Center for Drug Evaluation and Research.

Last, we classify the orphan approvals by therapeutic area, based on their orphan disease indication.

## Results

**Fig. 1:**
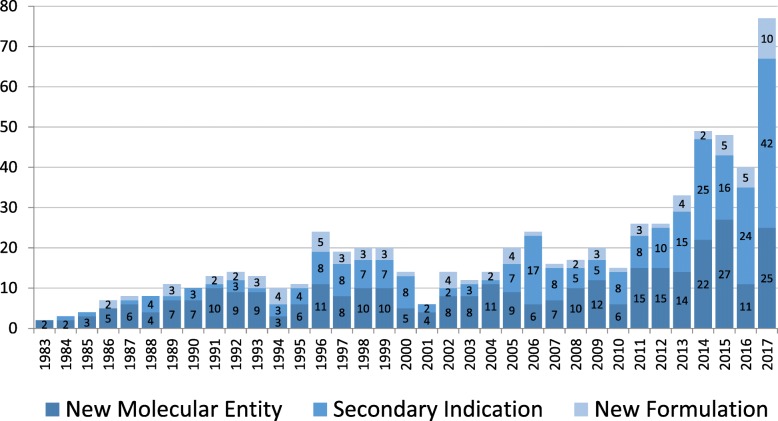
Orphan Drug Approvals 1983–2017, by regulatory approval type

We found a total of 667 orphan drug designated approvals. Approvals of orphan designated drugs have been increasing over time, with the largest number of approvals being seen in the most recent years, 2014—2017 (Fig. 1).

We also examine the types of orphan drugs being approved. NMEs account for the largest proportion of orphan product approvals, 49% (326). Secondary indications are the second highest category, constituting 39% (259) of the approvals.

Tracking these types of approvals over time, we find that in the most recent years, average number of NMEs approved has substantially increased. Additionally, much of the increase in rare disease approvals in the most recent years appears to be driven by the increase in the number of secondary indications being approved. In 2017 these approvals almost doubled from their previous high. New formulations appear to be consistent over time, although we also found a doubling of the previous high of these approvals in 2017.

**Fig. 2:**
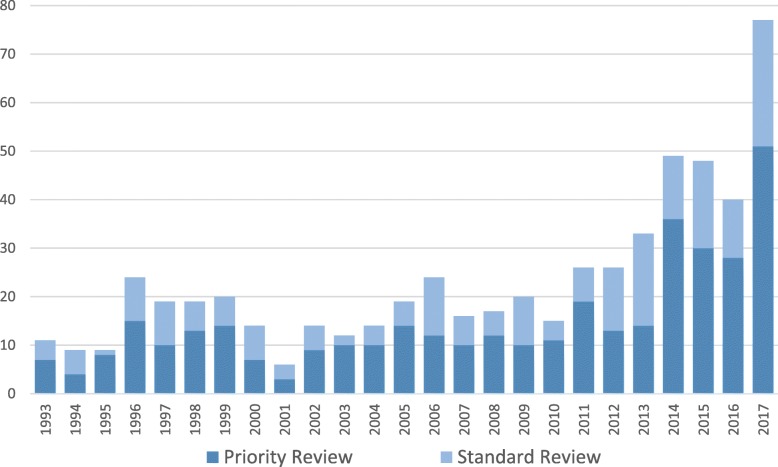
Orphan Drug Approvals 1993–2017, by priority vs standard review designation

We also examine the number of priority orphan approvals (as the priority review program does not span the entire ODA period, the data go back only to 1993 rather than 1983) (Fig. 2). In aggregate, of the 580 orphan approvals that occurred after the priority review program was enacted, 64% (369) were priority review, and 36% (211) were a standard review.

**Fig. 3:**
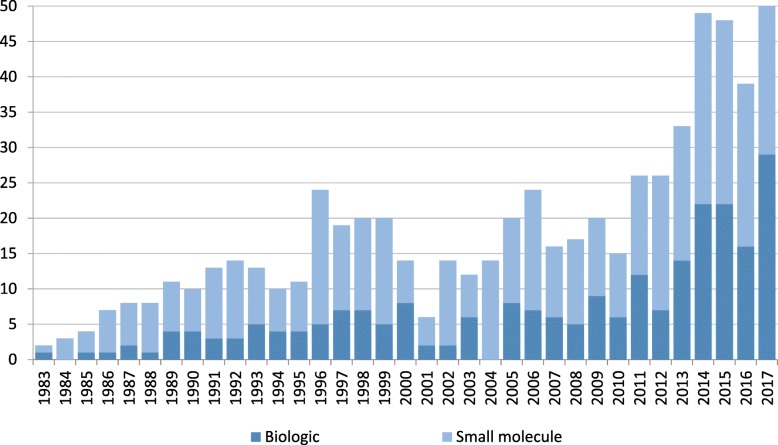
Orphan Drug Approvals 1993–2017, by drug versus biologic

The orphan approvals are also categorized by whether they were small molecule drugs or biologics. Over the study period, 36% (238) of the approved orphan drugs are biologics, and 64% (429) are small molecule drugs. While the most recent four years have seen a large increase in the number of biologic drug approvals for rare diseases, there is also an overall trend of increasing small molecule drugs over the entire study period (Fig. 3).

**Fig. 4:**
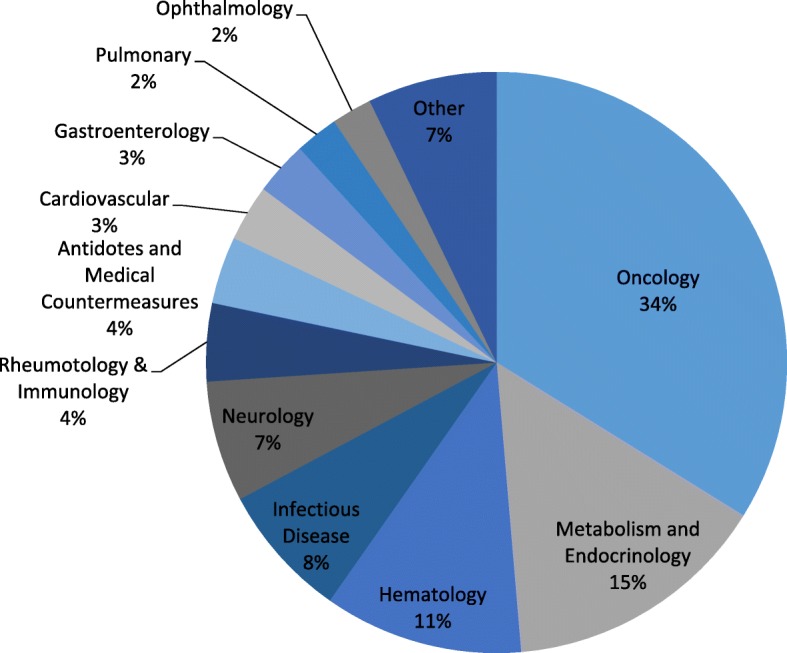
Orphan Approvals, by therapeutic area

The orphan approvals are most concentrated in three therapeutic categories: oncology (34%), metabolic and endocrine (15%), and hematology (11%) (Fig. 4). There has also been significant rare disease drug development in infectious diseases (7%), neurologic conditions (6%), and rheumatology and immunology (5%).

**Fig. 5:**
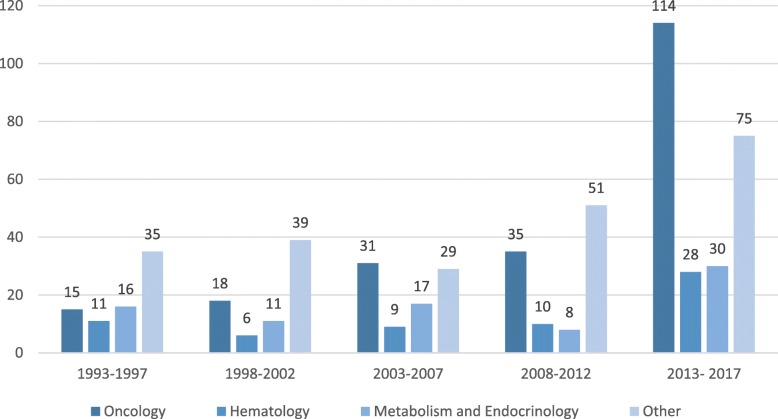
Orphan Approvals by therapeutic area, in 5-year periods

Additionally, while approval of oncology products has surged in the most recent five years, the increase does not come at the expense of approvals for other therapeutic areas (Fig. 5). This large spike in oncology products between 2013—2017, a total of 113 approvals, is primarily composed of secondary indications 53% (60), with NME approvals a distant second, at 39% (44). In comparison, in 2008—2012, only 35 oncology approvals occurred, with 51% (18) being NMEs and 40% (14) secondary indications.

**Fig. 6:**
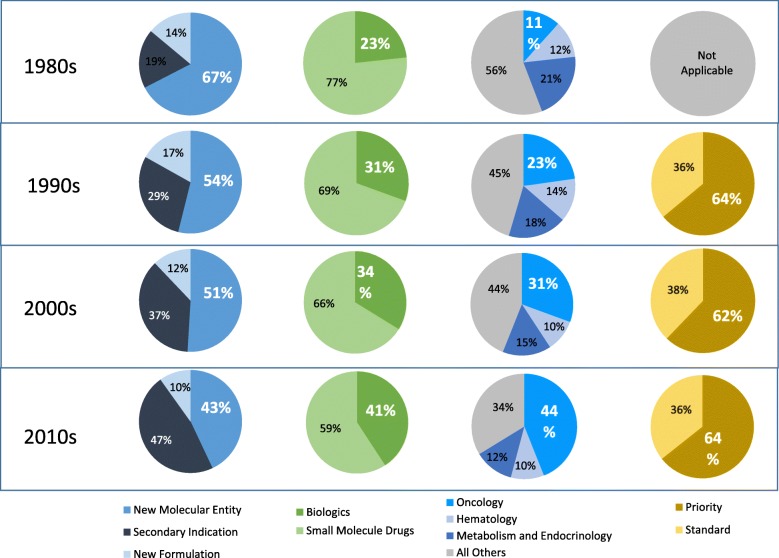
Orphan approvals, by decade, by multiple characteristics

Reviewing the data by overall trends, and year-by-year, provides important insights into orphan drug approvals. However, given that our data extends for more than thirty years, we are also able to provide graphic representation of how these trends have changed over the decades (Fig. 6). Specifically, we look at how the proportion of novel drugs approvals, biologics approvals, oncology approvals, and priority review drugs for rare diseases have changed.

We find that the proportion of new orphan drug approvals that are NMEs has decreased significantly, from over 67% in the 1980s to 43% in the 2010s. While the absolute number of NME approvals has increased over this timeframe, the reduction in the percentage share of NMEs has been driven by an even greater increase in the number of secondary indication orphan approvals.

Additionally, we find that there has been almost a doubling in the proportion of orphan approvals of biologic drugs between the 1980s and 2010s, from 23 to 41%. The trend for rare oncology products is even more distinct. While approvals for the second largest therapeutic category, rare metabolic and endocrine disorders, has decreased somewhat, the proportion of rare oncology approvals have quadrupled, from 11% of all approvals in the 1980s, to 44% in the 2010s.

Interestingly, the distribution of priority review versus standard review orphan drugs has remained stable over the study period. Priority reviews have accounted for approximately two thirds of all orphan drug approvals in each decade.

## Discussion

Overall, the results of our study indicate that the volume of orphan drug development continues to be strong, and approvals have even increased significantly in the most recent years. The recent growth in orphan approvals has been driven largely by two factors: an increase in the number of secondary indications being approved and the number of rare oncology products being approved.

The proportion of secondary indications being approved has more than doubled, from 19% of all approvals in the 1980s to 47% in the 2010s. As discussed in further detail below, this increase is driven largely by the increase in oncology approvals for secondary indications.

NME orphan drugs saw a significant proportional decrease during the study period, going from two thirds of total approvals to less than half. However, if we look at the absolute number of approvals, we can see that this change is not being driven by a decrease in the number of NME or new formulation approvals, but rather by the large increase in the number of secondary indication approvals; in fact, the trend in the number of NMEs being approved every year is increasing in recent years. We believe that this continued increase in orphan NME approvals signals a strong foundation for rare disease research, and that drug development in this space continues to be profitable.

While orphan NME approvals are an important part of rare disease drug development, the approval of secondary orphan indications is also a significant contribution to treating rare disease patients. The approval of a secondary indication means that safety and efficacy data—including information vital to clinicians, such as dosing or efficacy in pediatric subpopulations—has been systematically studied in that rare disease population. These are typically areas where patients often have little to no treatment options and may resort to taking medication off-label without the benefit of knowing the safety and efficacy of the drug in treating their particular disease or condition, so the addition of this information is an important public health contribution. Additionally, reimbursement is often more straightforward for approved indications than it is for the off-label use of the drug, which may improve patient access to therapies.

However, it is difficult to tell without further analysis whether these secondary indication approvals are true innovations, or simply extensions of existing product lines for business development [13]. It is clear that some types of drugs, including biologics and oncology products, which are well represented in orphan approvals, tend to have more label expansions than other types of drugs. But we are not able to determine the cause of this difference from the data at hand.

The increase in orphan approvals in recent years is also driven by the increase in the number of oncology drugs being approved. It appears that in the most recent five years, while orphan oncology NMEs have more than doubled, compared to approvals in the five preceding years, the number of secondary oncology indications has more than quadrupled. It is difficult to determine whether this amount of rare oncology drug development can be attributed to the ODA, or whether it is a by-product of the increases in basic science and translational research in oncology over the last few decades [4, 14, 15].

While this major increase in oncology approvals are the most dramatic, it is important to note that, we also see increases in approvals in other therapeutic areas. For example, in the most recent years, 2012—2017, there were higher numbers of approvals for the other areas studied: hematology, metabolic and endocrine, and a grouping of the other therapeutic areas. We believe that this implies that the incentives of the ODA aren’t being monopolized by a single area.

While not as drastic as the other results in this study, we also find that there has been a steady increase in the number of biologic products being approved for orphan indications. This trend may have significant implications for the prospect biosimilar entry for these drugs, which may affect affordability and therefore patient access. These products are very complex, and there are therefore higher costs to developing and manufacturing biosimilars: it is therefore possible that there may be more limited biosimilar entry of these products in rare disease markets than in large markets.

However, because the biosimilar pathway was only enacted by Congress in 2009, we have not yet had sufficient time to assess how biosimilar entry will evolve, in terms of competition and prices, especially in the rare disease space. Our finding that there is an increasing number of biologic orphan products being approved implies that, in the future, these unknowns regarding biosimilar entry in this space may become an important consideration for patients and payers in terms of future access and affordability of rare disease treatments.

Lastly, this study also provides evidence that the clinical value of orphan approvals has remained stable over time; even with the large increase of approvals in the most recent years, we have not seen a corresponding decline in the importance of these orphan drugs. We use the priority review designation as an indicator for products that may represent significant improvements in treatment for patients. A stable two thirds of all orphan drug approvals (excluding the pre-1992 period) have received priority review, suggesting that the majority of orphan drugs provide significant improvements over existing therapies. For many of these drugs, the rare diseases they are treating do not have any existing therapies, making these drugs even more critical to patients.

The purpose of this study is to provide a more nuanced understanding of the types of drugs that have been approved with an orphan designation, since the creation of the program in 1983. The results appear to indicate that the public health goals of this program are being met: no single therapeutic area or type of drug is being approved at the expense of the others. For example, while oncology products and secondary indication approvals are rising, neither appears to be displacing other types of approvals, which are rising as well.

However, these results can only highlight the successes of the ODA. This research cannot provide any illumination as to what types of drugs, if any, are not being developed, for reasons either for scientific (e.g. lack of understanding of the disease) or economic (e.g. trials too expensive to conduct, even with current incentives). Future research is needed to investigate this question further.

## Conclusion

In this study, we investigate the landscape of all types of orphan drug approvals between 1983—2017, including both orphan NME drugs and secondary orphan indications. We found that 667 distinct orphan drug approvals occurred during this period. The volume of these approvals has been increasing over time, especially in the last five years, but there has been no change in the level of innovativeness of these approvals, as measured by the proportion of priority review drugs. From these results, it appears that the ODA has been successful in stimulating the development of drugs for rare diseases over the past thirty years, and the effect is seen over multiple dimensions, including the types of approvals and therapeutic areas.

## Additional file

Additional file 1: Appendix: Approved Orphan Products Dataset. (XLSX 92 kb)

## Abbreviations

FDA: US Food and Drug Administration
NME: New molecular entity
ODA: Orphan Drug Act
